# Establishment of Haemonetics TEG 6s Reference Interval in Healthy Dogs

**DOI:** 10.1111/vcp.70104

**Published:** 2026-05-19

**Authors:** Kaylan Whetstone, Justin Heinz, Lonnie E. Grantham, Michaela Stavropoulos, Chelsie Hall, Floris C. Dröes, Thomas H. Edwards

**Affiliations:** ^1^ College of Veterinary Medicine, Texas A&M University College Station Texas USA; ^2^ US Army Institute of Surgical Research JBSA Fort Sam Houston Texas USA

**Keywords:** coagulation, reference interval, TEG 6s, viscoelastic monitoring

## Abstract

**Background:**

A thromboelastogram viscoelastic monitor (the TEG 6s) has recently become available for use in veterinary patients. It is therefore necessary to establish reference intervals for the new device in each species where it is employed. This study aimed to establish accurate and acceptable reference intervals for the TEG 6s in dogs.

**Objective:**

The goal of this study was to establish dog reference intervals (RIs) for the TEG 6s by sampling healthy adult dogs from a variety of breeds.

**Methods:**

One hundred and thirty dogs (130) aged 1–9 years old were enrolled in this study. Inclusion criteria included healthy adult dogs with no previously reported coagulopathies, no medications that are known to alter coagulation, and normal CBC, biochemistry, and physical exam. TEG 6s measurements were performed using a Haemonetics Global Hemostasis cartridge and citrated blood sample.

**Results:**

One hundred and twenty‐three (123) dogs were included in the TEG 6s RIs. The determined RIs for the citrated kaolin activated were reaction time (R) 2.1–5.6 min, kinetic time (K) 1.1–2.5 min, alpha angle 64.6°–74.9°, and maximum amplitude 47.3–62.0 mm. The determined RIs for citrated Rapid TEG (CRT) maximum amplitude and reaction for the kaolin‐heparinase activated were 40.0–59.3 mm and 2.1–5.6 min, respectively.

**Conclusions:**

RIs were established in a large group of normal dogs, which can assist small animal veterinarians with the interpretation of a newer viscoelastic testing method.

## 1 Introduction

Viscoelastic testing allows providers to obtain a global coagulation assessment, from clot formation to fibrinolysis, to assist in determining appropriate treatments for their patients. Human studies have evaluated the utility of viscoelastic test‐guided hemostatic resuscitation in both elective and emergent settings. Systematic reviews identified that viscoelastic testing can reduce length of stay, predict massive transfusion requirements, and decrease mortality compared to conventional coagulation testing. Additionally, this type of testing was helpful in blood product allocation and conservation [1, 2]. More recent studies highlight the importance of viscoelastic assessment in traumatized canines to document abnormalities in hemostasis. In one study of 33 traumatized dogs, rotational thromboelastometry (ROTEM) demonstrated hypocoagulability in a third of animals, with 6 dogs progressing to hypercoagulability within 24 h of treatment [3, 4]. A single veterinary study exists evaluating the utility of viscoelastic testing in guiding transfusion therapy in patients with various hemostatic abnormalities, ranging from coagulopathy to thrombocytopenia to disseminated intravascular coagulation. Utilizing viscoelastic testing in these cases was beneficial in guiding treatments. Although this study described only four cases, this small series supports the need for further studies evaluating viscoelastic‐guided therapies in veterinary medicine [4].

Recently, a viscoelastic monitor approved for use in people has been evaluated in dogs. The TEG 6s (Haemonetics, Boston, MA) is a cartridge‐based coagulation analyzer licensed for coagulation monitoring in people with trauma, among other indications [5]. TEG 6s has not yet been extensively researched in dogs, unlike its predecessor, the TEG 5000. Wheeler et al. established that the TEG 6s is not interchangeable with the TEG 5000, as the results from the two analyzers differ when blood is measured from the same dog at the same time. Additionally, the two analyzers use different methods to obtain hemostatic data [6]. Unlike the TEG 5000, the TEG 6s does not require any pipetting of solutions or blood, which reduces some user error. The TEG 6s system utilizes resonance‐frequency technology, combined with LED illumination, to obtain measurements of the blood sample throughout the coagulation process. Any changes in resonance are detected by the TEG 6s analyzer and converted to a graphical image. The TEG 6s performs thromboelastograms simultaneously, using roughly 0.5 mL of citrated anticoagulated blood and a disposable cartridge. The TEG 6s has the ability to run four different cartridges, which include Global Hemostasis, Global Hemostasis—HN, Global Hemostasis with Lysis, and PlateletMapping. The Global Hemostasis cartridge has four tracings, including intrinsic pathway activated kaolin, kaolin with heparinase, RapidTEG activated by kaolin and tissue factor, and functional fibrinogen, which uses abiximab and tissue factor. These tracings provide a total of eight parameters, including two reaction times (R), clot kinetics (K), alpha angle, three maximum amplitudes (MA), and the level of functional fibrinogen. This cartridge was designed to assess the risk of hemorrhage or thrombosis for patients as a bedside evaluation. The difference between the Global Hemostasis and the Global Hemostasis—HN cartridge is that the HN uses heparinase with RapidTEG and functional fibrinogen instead of tissue factor. This added heparinase enhances clinical decision‐making with known or suspected heparin in circulation. The Global Hemostasis with Lysis includes only three tracings: an intrinsic pathway‐activated kaolin assay (R and LY30%), RapidTEG, and functional fibrinogen. Lastly, the PlateletMapping cartridge, which has four tracings to determine overall platelet clot strength, fibrin‐only clot strength, and platelets' response to adenosine diphosphate and arachidonic acid [7, 8, 9]. This analyzer has great potential for use in any environment because it is compact and lightweight, utilizes reagents that can be stored at room temperature, disposable cartridges, and is not subject to vibrations, unlike its predecessor [5, 7, 10].

Recently, another study established a canine reference interval for the TEG 6s using 40 healthy dogs [11]. This reference interval has already been used in a study evaluating the hemostatic profile in dogs with chronic enteropathies to determine if the dogs were hypercoaguable [12]. ASVCP reference interval guidelines emphasize the need for larger sample sizes to ensure that the intervals accurately reflect true population characteristics and minimize statistical errors associated with small populations [13]. This study aims to establish a reference interval for the Haemonetics TEG 6s using a more robust population of > 120 healthy dogs. We hypothesized that we could generate acceptable reference intervals for the TEG 6s in dogs.

## 2 Materials and Methods

### 2.1 Ethics Approval

This study was performed under an approved International Animal Care and Use Committee (IACUC) protocol at Texas A&M University. Signed owner consent was obtained before all sample collections.

### 2.2 Animals and Sample Collection

Canine volunteers were recruited from faculty, staff, and students at the Texas A&M University College of Veterinary Medicine & Biomedical Sciences (College Station, Texas, USA) during the months of December 2024 to February 2025. The study used the following inclusion criteria: dogs aged 1–9.9 years old who weigh greater than 11 pounds (5 kgs), not on any medications (e.g., Trazodone) that alter their ability to form clots, have not received a transfusion prior to enrollment, and are apparently healthy based on owner history and physical examination. One hundred and thirty (130) apparently healthy individuals were evaluated via a history, a physical exam by a veterinarian, a standard complete blood count, and a standard serum biochemistry profile. If an abnormality on history or physical exam was discovered, the individual would not be enrolled in the study. If a significant abnormality on complete blood count or serum biochemistry was discovered, the individual's data were excluded from the study. Exclusion criteria for the complete blood count included anemia (i.e., hematocrit < 35.0%), thrombocytopenia (i.e., platelet count < 100 000/μL), and leukopenia (i.e., < 4 × 10^3^/μL), or leukocytosis (i.e., > 16 × 10^3^/μL). A blood smear was evaluated when less than 100 000/μL platelet counts were noted on the CBC. Exclusion criteria for the serum biochemistry included > 25% elevations in liver values (alanine transaminase, alkaline phosphatase, total bilirubin), kidney values (blood urea nitrogen, creatinine), or significantly deranged electrolytes.

From each dog, 5 mL of whole blood was collected from the cephalic or lateral saphenous vein using a 20‐gauge butterfly needle and two 3 mL syringes. The venous blood was split in the following order: 2.7 mL in a 3.2% sodium citrate tube, followed by 1 mL in a clot tube with no additives, using a fresh 20‐gauge needle to pierce the tops. No waste sample was obtained prior to filling the tubes. A TEG 6s Global Hemostasis cartridge (Haemonetics, Braintree, MA, USA) was placed in the machine, and once the sample was loaded according to the manufacturer's instructions, the test was automatically initiated. This cartridge uses kaolin, kaolin with heparinase, rapid‐TEG (kaolin plus tissue factor), and functional fibrinogen using abciximab to evaluate the global hemostasis of the patient. Quality control was performed on the TEG 6s per the manufacturer's specifications. Internal controls were performed on startup of the analyzer and upon insertion of each cartridge. A reagent quality control was performed upon receipt of each batch of cartridges. A complete blood count was performed on a ProCyte Dx Hematology Analyzer (IDEXX, Westbrook, Maine, USA) using the remainder of the sodium citrated sample. TEG 6s and CBC analysis were performed within 20 min of blood sample collection. The clot tube was centrifuged within 1 h of collection, and the serum was extracted and stored at −80°C until analyzed for the serum biochemistry panel. Samples for biochemical testing were analyzed on a Beckman AU480 (Brea, CA, USA).

### 2.3 Statistical Analysis

Reference intervals for each analyte were calculated using statistical approaches recommended by the American Society for Veterinary Clinical Pathology (ASVCP) Quality Assurance and Laboratory Standards Committee. This study did not consider functional fibrinogen or estimated functional fibrinogen level in its statistical analysis because it utilizes abciximab to inhibit platelet function. Outlier identification was performed by Horn's algorithm using Tukey's interquartile (IQ) fences, which identifies multiple outliers located at the upper and lower extremities. Graphs of individual data points were reviewed to inspect extreme values. Suspected outliers were removed if deemed biologically implausible, analytically erroneous, or if individuals on the TEG 6s had multiple values at the upper or lower end of the readable range of the machine. The analysis was then repeated with the revised dataset. All data from all dogs that were deemed healthy and met our inclusion and exclusion criteria were included. All values, including those that were flagged as potential outliers by the IQR method, were included in the reference interval calculations. All analytes had a minimum of 122 valid reference individuals, thus allowing for calculation of the central 95% of values for nonparametric reference intervals and 90% confidence intervals of the upper and lower reference limits. All statistical analyses were performed using SAS software Version 9.4 TS Level 1M8 of the SAS System for Windows (SAS Institute, Cary, NC, USA).

## 3 Results

A total of 123 client‐owned dogs (62 spayed females, 3 intact females, 57 castrated males, and 1 intact male), with a median age of 4 years and 6 months (range 1–9.9 years), were enrolled. The breeds represented included mixed breed dogs (49), Australian shepherds (13), Labrador retrievers (10), poodles (7), pit bull terriers (4), golden retrievers (5), border collies (4), 3 of each Rhodesian ridgeback, Doberman pinscher, German shepherd dog, 2 of each Australian cattle dog, pug, coonhound, great Dane, Belgian Malinois, Jack Russell terrier, and one of each of the following: dachshund, chihuahua, dalmatian, Boykin spaniel, Cavalier King Charles spaniel, Dutch shepherd, Dogo Argentino, Bernese mountain dog, Dandie Dinmont terrier, and great Pyrenes.

All included dogs were determined to be healthy based on history, physical exam, CBC, and serum biochemistry panel. Five dogs were initially excluded due to significant abnormalities in either the chemistry or CBC data. Of the CBCs, one was excluded due to a high white blood cell count, another due to a low white blood cell count, and one was excluded due to low platelets. Two dogs were excluded due to elevated ALT. During review of TEG 6s thromboelastography data, 3 additional dogs were removed due to multiple assays out of the range of the machine. The citrated kaolin (CK) reaction time (R) value for one spayed female was excluded due to out‐of‐range value. The CK kinetic (K) and CK alpha angle (which are correlated) values for another spayed female were excluded due to out‐of‐range values. All other values, including those that were flagged as potential outliers by the IQR method, were included in the reference interval calculations. This resulted in the data from 122 dogs available for analysis on R, K, and alpha angle, and data from 123 dogs available on maximum amplitude CK, maximum amplitude Rapid TEG (CRT), and citrated kaolin‐heparin activated (CKH) R (Table 1). The reference intervals established in this study for the CK activation in dogs are as follows: R 2.1–5.6 min, K 1.1–2.5 min, Alpha Angle 64.6°–74.9°, and Maximum Amplitude 47.3–62.0 mm. The reference interval this study established for the CRT maximum amplitude is 40.0–59.3 mm, and for the reaction for the CKH is 2.1–5.6 min. Scatter plots were generated for each analyte to visualize the distribution and identify potential outliers. The majority of data points were evenly distributed without clustering, with a few outliers identified. The plots visually reinforce that the reference intervals accurately represent the range of results observed in this healthy dog population (Figures 1 and 2).

**TABLE 1:** Reference intervals for point‐of‐care viscoelastic test (TEG 6s) variables and 90% CIs of upper and lower RIs (*n* = 123 healthy dogs) following ASVCP QA and LCS guidelines by using nonparametric methods.

Measurement	Units	*n*	Mean	SD	Median	Min	Max	LRL of RI	URL of RI	CI 90% of LRL	CI 90% of URL
Reaction—CK	minutes	122	4.12	0.97	4.20	1.3	6.8	2.1	5.6	1.3–2.4	5.4–6.8
Kinetic—CK	minutes	122	1.75	0.36	1.80	1.0	3.1	1.1	2.5	1.0–1.2	2.3–3.1
Alpha angle—CK	degrees	122	70.05	2.74	70.15	58.7	75.6	64.6	74.9	58.7–66.5	74.7–75.6
Maximum amplitude—CK	mm	123	54.41	4.09	54.60	42.5	64.5	47.3	62.0	42.5–48.5	60.8–64.5
Maximum amplitude—CRT	mm	123	50.20	5.45	50.40	40.0	63.1	40.0	59.3	40.0–40.0	59.1–63.1
Reaction—CKH	minutes	123	3.95	0.93	4.10	1.3	5.7	2.1	5.6	1.3–2.4	5.4–5.7

Abbreviations: CI, confidence interval; CK, citrated kaolin; CKH, citrated kaolin‐heparin; CRT, RapidTEG; LRL, lower reference limit; RI, reference interval; SD, standard deviation; URL, upper reference limit.

**FIGURE 1:**
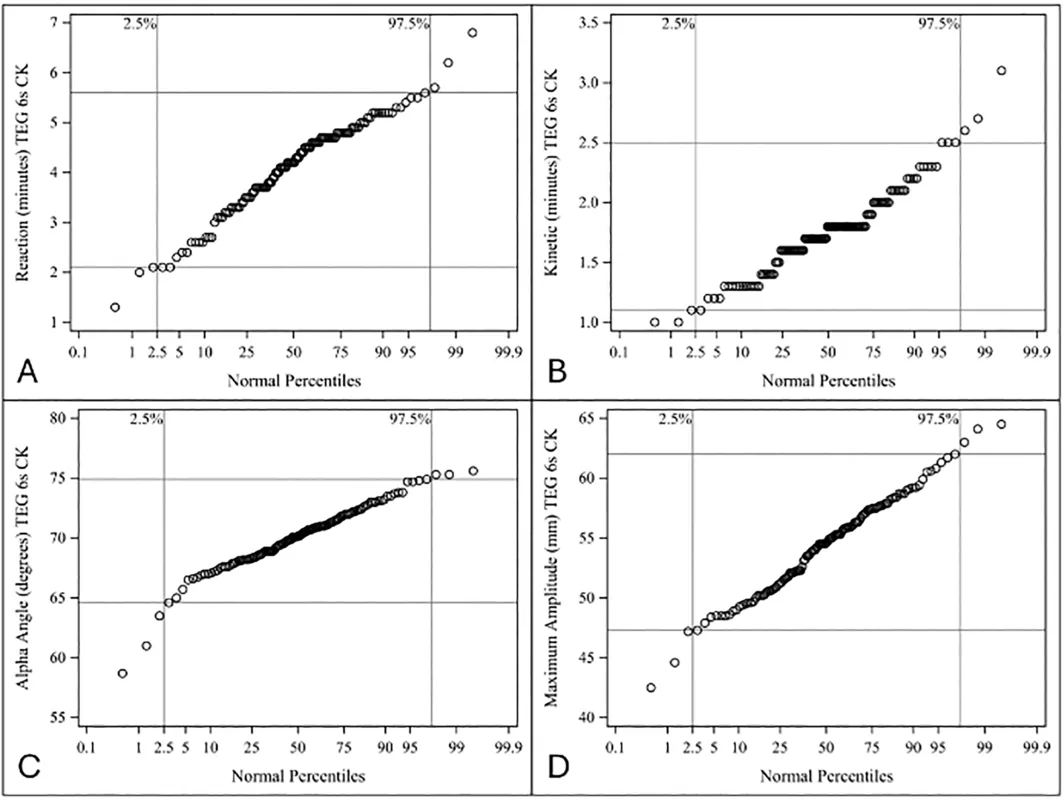
Scatter plots of the citrated kaolin (CK) assays, including (A) reaction time (min), (B) kinetic time (min), (C) alpha angle (degrees), and (D) maximum amplitude (mm), to illustrate the distribution of values in 123 healthy dogs.

**FIGURE 2:**
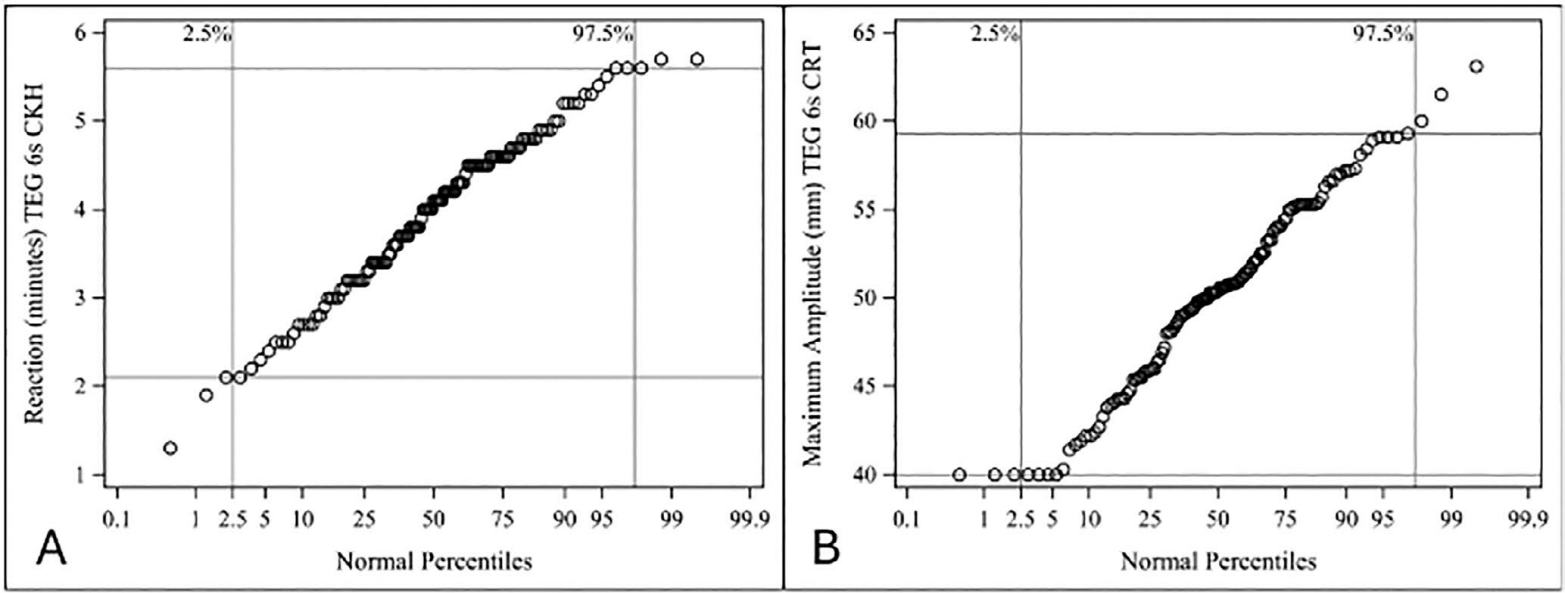
Scatter plots of (A) citrated kaolin‐heparin (CKH) reaction time and (B) maximum amplitude of rapid TEG to illustrate the distribution of values in 123 healthy dogs.

## 4 Discussion

This study establishes the reference intervals for the Haemonetics TEG 6s global hemostasis cartridge using a robust population of healthy adult dogs. A total of 123 dogs were included, representing 23 breeds, including mixed‐breed dogs which provided a realistic representation of the population of commonly owned breeds seen in veterinary medicine. Any breed of dog was eligible to participate in this study; thus, no breeds were explicitly included or excluded, such as Greyhounds. If a particular breed is not included, it is due to the availability of the canine volunteers within the study's location. The number of samples obtained in this study is in keeping with the ASCVP guidelines for an accurate and reliable estimate of the reference limits [7]. As sample size increases, the confidence intervals around the reference limits become narrower, indicating a greater certainty about the true range of values in a healthy population. Overall, very few outliers were identified from this population. When subjects with outliers were identified in multiple parameters from the analyzers, all of the subject data were removed from our analysis. When one parameter from our apparently healthy population was flagged, this data remained in our analysis so as not to bias our data by removing certain datapoints post hoc. Because of this, we believe the data contained here represents statistically acceptable RIs for all parameters.

The reference intervals identified in our study were similar to those previously described by Wilkinson et al. but overall provided a narrower interval. The reference interval for the CKR value in the previous study was lower than the results reported here. CKK, CK Alpha Angle, CK MA, and CRT values, while similar, had a wider interval. The reason for the difference in the reference intervals likely arises from the number and breed of dogs enrolled to create these reference intervals. The previous study included 40 healthy dogs as the control group, with 13 breeds represented. Larger participant numbers/sample size allows for more certainty in the estimation of the RI [7]. Wilkinson et al. and Barth et al. included the functional fibrinogen test in their results and Barth et al. noted that 82% of the results significantly exceeded the upper limit indicating hypercoagulability [11, 12]. This study intentionally did not include functional fibrinogen (FF) or estimated functional fibrinogen level (FLEV). This assay is designed to isolate fibrinogen's contribution to clot strength by inhibiting platelet function using abciximab [6]. Abciximab used in the TEG 6s has previously been shown to be unsuitable for inhibiting platelet function in canine blood, and therefore, we did not report on the results of this assay or the results of the estimated functional fibrinogen level [14]. Abciximab is a monoclonal antibody that targets human platelet glycoprotein IIb/IIIa receptors and does not effectively inhibit canine platelets. Brainard et al. found that adding abciximab to canine citrated blood did not affect the TEG tracing [14]. The estimated functional fibrinogen level (FLEV) is derived from the maximum amplitude of the functional fibrinogen on the TEG assay. Because the functional fibrinogen assay in dogs is not reliable, it is assumed that the FLEV value is also not reliable.

The TEG 6s is poised to replace the current standard TEG 5000 for clinical use in humans as the TEG 5000 is expected to be phased out of production. Wheeler et al. previously established that the TEG 6s results are not interchangeable with the TEG 5000, prompting this study to establish reference intervals specifically for the TEG 6s. The likely reason for this discrepancy is the different technology used within the analyzers and the physical properties of the TEG 6s cartridge and the TEG 5000 cup and pin. The TEG 5000 uses an oscillating cup and a suspended pin to obtain a clotting measurement, allowing different reagents to be added during the assay. The motion of the oscillating cup is transmitted to the pin, the device measures the torque on the pin, and the magnitude of the pin's motion is directly related to clot strength. The TEG 6s utilizes resonance‐frequency technology, combined with LED illumination, to obtain measurements of the blood sample throughout the coagulation process. These different mechanisms likely resulted in the different results and reference intervals seen between the two devices.

There are some limitations to this current study. Dogs were considered healthy based on their lack of significant medical history and unremarkable physical examination results. None of the dogs underwent imaging to detect subclinical diseases that could have influenced the hemostatic state of some animals. This study only included healthy dogs and did not assess unhealthy or ill individuals or those with known coagulopathies. When obtaining the blood from the participants, a waste sample was not obtained due to the size of some of the patients, in an attempt to mimic clinical settings. There has been some dispute in the literature about the necessity of a discard tube prior to coagulation testing, with more recent literature indicating this practice is likely not necessary [15]. Blood smears were performed only on the individuals who had platelets below the reference interval. This doesn't account for any discrepancies within the normal platelet count of individuals. Some dogs had TEG 6s parameters at the upper or lower limits of the reportable values specific to the machine. When this happened, the limit was reported. Any biologically implausible data were excluded because they likely reflected measurement errors rather than true physiological values in healthy dogs. One limitation of the TEG 6s Global Hemostasis cartridge is the absence of fibrinolysis parameters such as LY30%. Although the TEG 6s offers a cartridge capable of assessing fibrinolysis, it lacks the comprehensive data provided by the Global Hemostasis cartridge used for the citrated kaolin assay, as it only measures reaction time and lysis [8]. Without the ability to evaluate clot kinetics and maximum amplitude, clinicians are limited in their assessment of the intrinsic pathway and overall coagulation dynamics. The Global Hemostasis with Lysis cartridge is well‐suited for evaluating trauma patients, where fibrinolytic activity plays a greater role; however, it lacks the necessary components to fully characterize coagulation in non‐traumatic patients with potential coagulopathies. Additionally, while a complete trace is available in the kaolin and rapid TEG‐activated assays, numerical values are not displayed on the home screen, which may hinder rapid interpretation. Future studies are warranted to validate the performance of the platelet mapping and fibrinolysis cartridges and to establish appropriate reference intervals for these assays in dogs. The data collected from TEG 6s in this study were not cross‐referenced with a validated test, such as VCM.

Further studies are indicated to evaluate correlations between TEG 6s variations and other viscoelastic testing parameters. The TEG 6s is poised to replace the current standard TEG 5000 for clinical use in humans as the TEG 5000 will be phased out of production. The RIs provided will assist small animal veterinarians with the interpretation of canine viscoelastic testing results regarding the TEG 6s.

## Funding

This study is funded by a grant from the Department of Defense.

## Conflicts of Interest

The authors declare no conflicts of interest.
